# Optimizing Sowing Time and Density Can Synergistically Improve the Productivity and Quality of Strong-Gluten Wheat in Different Ecological Regions of Shandong Province

**DOI:** 10.3390/plants14030372

**Published:** 2025-01-26

**Authors:** Guangzhou Chen, Weibao Yu, Yushen Zheng, Le Zhang, Jisheng Si, Kainan Zhao, Ruochen Li, Deqiang Zhao, Lei Qu, Bin Zhang, Shengdong Li, Lingan Kong, Zaidong Yang, Huawei Li

**Affiliations:** 1 Crop Research Institute, Shandong Academy of Agricultural Sciences, Jinan 250100, China; 2 College of Agronomy, Xinjiang Agricultural University, Urumqi 830052, China; 3 College of Agronomy and Biotechnology, China Agricultural University, Beijing 100193, Chinab20213010007@cau.edu.cn (R.L.); zhaodeqiang1995@163.com (D.Z.); 4 Shandong Luyan Agricultural Variety Co., Ltd., Jinan 250100, China; qushitou-123@163.com

**Keywords:** sowing date, planting density, ecological region, yield, quality

## Abstract

Timely sowing is a crucial cultivation practice for enhancing crop productivity. In Shandong Province, inadequate supporting cultivation techniques are the primary factors limiting the yield and quality improvement of high-quality strong-gluten wheat (*Triticum aestivum* L.). A promising strategy for achieving synergistic improvements in both yield and quality involves matching the sowing date and density to the specific ecological conditions of each region. To explore this approach, we conducted continuous field experiments at three testing stations—Jining, Dezhou, and Yantai—across the major wheat-growing regions of Shandong Province from 2019 to 2021. Four sowing dates (T1: October 5; T2: October 15; T3: October 25; and T4: November 5) and seven planting densities (ranging from 135 × 10^4^ plants ha^−1^ to 405 × 10^4^ plants ha^−1^, denoted as D1–D7) were tested at each location. The results revealed that the wheat yield in each ecological zone initially increased, then decreased as the sowing dates were delayed. In Jining and Dezhou, high grain yields were typically observed at all densities under T3, while Yantai showed optimal yields under T2. Specifically, Jining achieved the highest grain yield of 9326.6 kg ha^−1^ with 315 × 10^4^ plants ha^−1^ on October 25 (T3D5), while Dezhou and Yantai reached their maximum yields under 225 × 10^4^ plants ha^−1^ on October 15 (T2D3), with yields of 8784.0 kg ha^−1^ and 9366.3 kg ha^−1^, respectively. Except in Dezhou, the wheat quality compliance rate at all sites followed an increasing trend initially, which then declined with later sowing dates. In Jining and Yantai, high-quality compliance rates were most frequently achieved under T2, while Dezhou showed optimal quality rates under T1. In conclusion, selecting appropriate sowing dates and densities can lead to synergistic improvements in both grain yield and quality of strong-gluten wheat across Shandong’s wheat-growing regions.

## 1. Introduction

The global demand for food is undergoing significant shifts due to rapid population growth and changes in dietary patterns [1,2]. As one of the world’s primary food crops, wheat (*Triticum aestivum* L.) supplies approximately 20% of the global energy and 22% of the protein in the human diet [3]. By 2030, global wheat consumption is expected to rise from 749 million tons (average level from 2018 to 2020) to 835 million tons (OECD-FAO, 2021). However, agricultural production is facing unprecedented challenges due to increased resource inputs, diminishing arable land, water scarcity, and the intensification of extreme weather events [4].

In China, wheat is a staple crop, with both its planting area and grain yield ranking among the highest globally. Given the limited farmland, the annual yield increase rate of crops needs to reach at least 2% to meet the growing domestic demand [5]. Wang et al. [6] predicted that China’s wheat demand will peak at 143 million tons by 2035, while the potential increase between 2020 and 2050 will be limited to 48 million tons. Meanwhile, with the rapid development of economy and agricultural reforms, the demand for wheat in China is gradually shifting from quantity to quality. Despite meeting the overall wheat demand, China still imports approximately 4 million tons of high-quality wheat annually, primarily strong-gluten and weak-gluten wheat. This reveals a structural gap between the production of high-quality wheat and the needs of the food industry [7]. Therefore, improving the production of high-quality wheat is essential for enhancing both grain yield and quality, which is critical for advancing agricultural reforms and improving food security in China.

The sowing date and planting density are key factors influencing both grain yield and quality in wheat production. These are also among the simplest, most precise, and environmentally sustainable agricultural practices [8]. By adjusting the sowing date, the utilization of ecological factors such as temperature, light duration, and rainfall can be optimized to ensure better synchronization between crop growth and local environmental conditions [9,10]. Planting density affects individual plant growth and development, as well as population size, which in turn impacts the efficiency of light energy utilization and material accumulation in crops [11,12]. Together, sowing date and planting density interact to shape wheat growth, material accumulation, and transport processes, ultimately influencing grain yield and quality.

Recent advances in breeding technology have led to the development of high-yielding and high-quality strong-gluten wheat varieties. However, the lack of clear recommendations for optimal sowing dates and densities in different ecological zones has hindered the full realization of their yield and quality potential, leading to inconsistent and unstable outcomes. We hypothesized that the optimal planting density for different sowing dates across experimental sites can enhance wheat yield and quality by improving the crop’s photosynthetic capacity and dry matter assimilation. Therefore, this study investigated the effects of different sowing dates and planting densities on wheat growth, photosynthetic production, and material accumulation in various regions of Shandong Province. The objectives of this study were to (1) assess the effects of sowing dates and planting densities on wheat growth and development, photosynthetic production, and material accumulation across different regions; (2) identify the variations in sowing dates and densities that synergistically improve both the grain yield and quality of high-quality strong-gluten wheat in Shandong Province’s ecological regions; and (3) determine the optimal sowing dates and planting densities for each ecological zone in Shandong. The findings of this study will provide technical guidance and theoretical support for developing high-yield, high-quality cultivation strategies for strong-gluten wheat in Shandong Province.

## 2. Materials and Methods

### 2.1. Experimental Site Description

**Figure 1:**
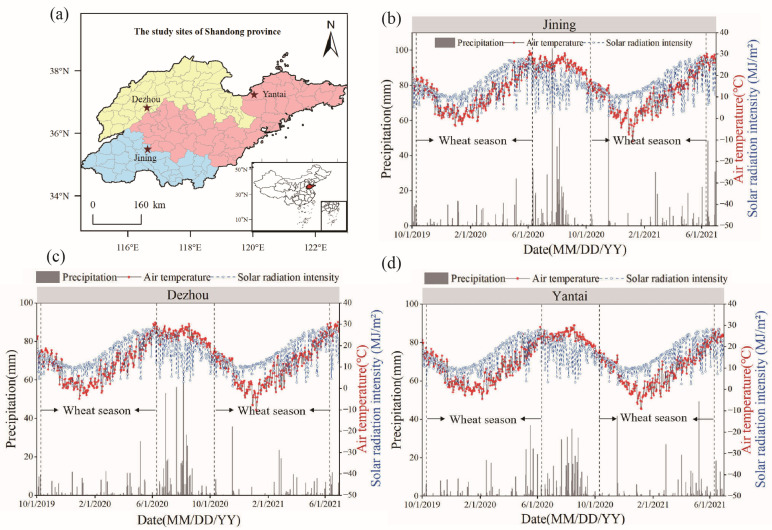
Overview of (**a**) and meteorological data for (**b**–**d**) the three experimental ecological sites in Shandong province. Note: The yellow area on the map represents the plain area of northwest Shandong; blue represents the lake and depression area in the southwestern plain of Shandong; and the pink area is the mountainous and hilly area of eastern and central Shandong.

This experiment was conducted from 2019 to 2021 across three sites in Shandong Province: Huaji Village, Jiaomiao Town, Qihe County, Dezhou City (36.640° N, 116.440° E); Dananpu Village, Da’an Town, Yanzhou District, Jining City (35.593° N, 116.773° E); and the Laizhou 8th experimental field, Xiyou Town, Laizhou City, Yantai City (37.177° N, 119.942° E). These locations represent distinct wheat-growing regions: the northwest Shandong plain, the southwest Shandong plain and lake basin, and the eastern and central Shandong mountainous and hilly areas (Figure 1a). The accumulated temperature (≥0 °C) during the wheat growing seasons of 2019–2020 and 2020–2021 was 2419.6 °C and 2299.2 °C in Huaji Village, 2333.3 °C and 2249.5 °C in Dananpu Village, and 2277.8 °C and 2189.0 °C in the Laizhou 8th experimental field. Rainfall during these periods was 200.0 mm and 264.5 mm in Huaji Village, 198.3 mm and 181.8 mm in Dananpu Village, and 290.0 mm and 293.3 mm in the Laizhou 8th experimental field. The solar radiation intensity was 3439.7 MJ m^−2^ and 3351.6 MJ m^−2^ in Dananpu Village (Figure 1b), 3412.2 MJ m^−2^ and 3295.9 MJ m^−2^ in Huaji Village (Figure 1c), and 3589.3 MJ m^−2^ and 3479.7 MJ m^−2^ in the Laizhou 8th experimental field (Figure 1d). The cultivation system in each zone follows a double-cropping annual pattern, with winter wheat and summer maize. The soil properties of the top 0–20 cm layer at each experimental site are presented in Table 1.

### 2.2. Experimental Design and Field Management

The high-yielding, high-quality, and strong-gluten wheat variety Jimai 44, recommended for Shandong Province, was selected as the experimental material for this study. The experiments were conducted for two consecutive years (2019–2021) at experimental sites across three ecological zones in Shandong Province. A randomized block design was adopted in the experiments. Four sowing dates were used: October 5 (T1), October 15 (T2), October 25 (T3), and November 5 (T4). For each sowing date, four planting densities were tested. In line with the strategy of increasing the density with later sowing, the planting densities were as follows: T1—135 × 10^4^ plants ha^−1^ (D1), 180 × 10^4^ plants ha^−1^ (D2), 225 × 10^4^ plants ha^−1^ (D3), and 270 × 10^4^ plants ha^−1^ (D4); T2—D2, D3, D4 and 315 × 10^4^ plants ha^−1^ (D5); T3—D3, D4, D5 and 360 × 10^4^ plants ha^−1^ (D6); T4—D4, D5, D6 and 405 × 10^4^ plants ha^−1^ (D7). This resulted in 16 treatment combinations, each replicated three times in an area of 6 m × 20 m. The experimental treatment combinations and abbreviations are summarized in Table 2. The fertilizer application consisted of 120 kg N ha^−1^, 60 kg P_2_O_5_ ha^−1^, and 120 kg K_2_O ha^−1^ prior to sowing, with an additional 120 kg N ha^−1^ applied at the jointing stage. Irrigation was performed twice—during the overwintering and jointing stage—with 600–750 m^3^ ha^−1^ of water applied each time, as measured by a water meter. After the previous maize harvest, straw was fully crushed and returned to the field, with deep plowing performed before sowing. Other field management measures including pest and weed control followed standard protocols for high-yield fields.

### 2.3. Sampling and Measurements

#### 2.3.1. Leaf Area Index, Photosynthetic Potential, and Net Assimilation Rate

Leaf area index (LAI, m^2^ m^−2^) was measured following the method described by Zhang et al. [13]. The mean leaf area index (MLAI, m^2^ m^−2^) was calculated as the average value of LAI during the entire growth period. Leaf Photosynthetic Potential (LAD, m^2^ d m^−2^) was calculated using Equation (1) [14].

LAD = MLAI × D(1)

where MLAI is the mean leaf area index (m^2^ m^−2^) and D is the effective number of days during the growth period (d).

The mean net assimilation rate (MNAR, g m^−2^ d^−1^), which reflects the increase in dry matter per unit leaf area per unit time, was calculated using Equation (2) [15].

MNAR = [(lnLA_2_ − lnLA_1_)/(LA_2_ − LA_1_)]×[(W_2_ − W_1_)/(t_2_ − t_1_)] (2)

where t_1_ and t_2_ are the time of two measurements (previous and next time); W_1_ and W_2_ are the dry weight at t_1_ and t_2_; LA_1_ and LA_2_ are the leaf area at t_1_ and t_2_; and ln is the natural logarithm.

#### 2.3.2. Yield and Related Indicators

At maturity, the number of ears in two representative rows (2 m long) of each plot was counted, and the number of ears per hectare was then calculated. A total of 30 consecutive individual plants were randomly selected from each plot and the number of grains per spike and thousand grain weight were measured. A 4 m^2^ sample from each plot was harvested to determine the biomass yield and grain yield after threshing. All harvested grains were left to fully ripen for two months before quality testing. The harvest index (HI) was calculated using Equation (3) [16].

HI = GY/BY(3)

where GY is the grain yield (kg ha^−1^) and BY is the biomass yield (kg ha^−1^).

#### 2.3.3. Quality Parameters

Grains that had completed the ripening process were ground into flour using a Swiss Breuer grinder. According to GB/T 5498-2013 [17], an HGT-1000 grain bulk density tester (Beijing Jingcheng Huatai Instrument Co., Ltd, Beijing, China) was used to measure the grain bulk density. The Kjeldahl method was used to determine the nitrogen content of the grains. The protein content of the grains was calculated by multiplying the nitrogen content by 5.7, and then converted it to a protein content with a 14% moisture content. According to the method GB/T 5506.2-2008 [18], the wet gluten content was determined using a Perten Boten GM2200 gluten washing instrument (Botong Ruihua Scientific Instruments (Beijing) Co., Ltd, Beijing, China). According to GB/T 14614-2019 [19], a German Brabant AT automatic powder quality analyzer (Brabender, Duisburg, Germany) was used to detect the dough’s powder quality parameters.

The quality parameters were normalized according to GB/T17892-1999 [20] and GB/T17320-2013 [21] (https://std.samr.gov.cn/gb/search/gbDetailed?id=71F772D7FF89D3A7E05397BE0A0AB82A (accessed on 2 January 2022)). When the value of a quality parameter meets or exceeds the strength standard, the probability (Xi) of the strength of meeting the standard is assigned as 100%. If the value is below the strength standard, the likelihood of meeting the standard is calculated using Equation (4):

Xi = actual value/strength annotation value × 100%(4)

The overall likelihood of the sample meeting the standard is the product of the compliance rates of the individual quality parameters.

### 2.4. Statistical Analysis

This study used WPS Office for data organization. SPSS 22.0 (SPSS Inc., Chicago, IL, USA) was used for performing the descriptive statistics, significance analysis, and correlation analysis. A one-way analysis of variance (ANOVA) was used to test the significant differences in the measured indicators among planting densities treatments under the same experiment site and the same sowing date. Average comparisons between treatments were performed using the least significant difference test at the level of *p* < 0.05. A regression analysis was performed to quantify the effects of combined sowing dates and planting densities on the grain yield. ArcGIS 10.2 (Environmental Systems Research Institute, Redlands, CA, USA) was used to make distribution maps of the three ecological zones. Origin 2021 (Origin Lab, Northampton, MA, USA) was used for creating the bar charts, scatter plots, and correlation plots.

## 3. Results

### 3.1. Leaf Assimilation Capacity

#### 3.1.1. Leaf Area Index

**Figure 2:**
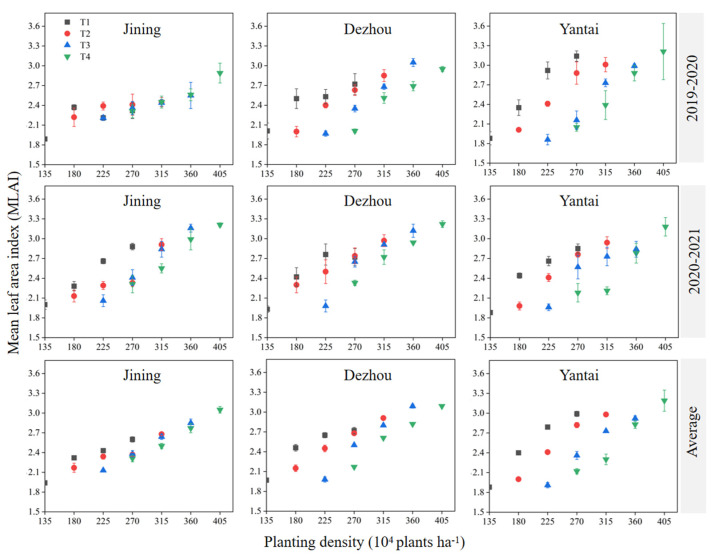
Effect of sowing time and density on mean leaf area index. Note: T1, T2, T3, and T4 indicate sowing on October 5, October 15, October 25, and November 5, respectively. Data are expressed as the mean of three replicates and vertical bars indicate standard deviation.

The average results from the three experimental sites over two years revealed that the mean leaf area index (MLAI) decreased as the sowing date was delayed at the same planting density. Conversely, the MLAI increased with higher planting densities for the same sowing date. Specifically, planting 405 × 10^4^ plants ha^−1^ on November 15 (T4D7: 3.11) resulted in a higher MLAI than planting 360 × 10^4^ plants ha^−1^ on October 25 (T3D6: 2.95), which was higher than planting 315 × 10^4^ plants ha^−1^ on October 15 (T2D5: 2.86), which, in turn, was higher than planting 270 × 10^4^ plants ha^−1^ on October 5 (T1D4: 2.77) (Figure 2). These findings suggest that increasing the planting density had a more significant effect on enhancing the MLAI than sowing earlier.

#### 3.1.2. Photosynthetic Potential

**Figure 3:**
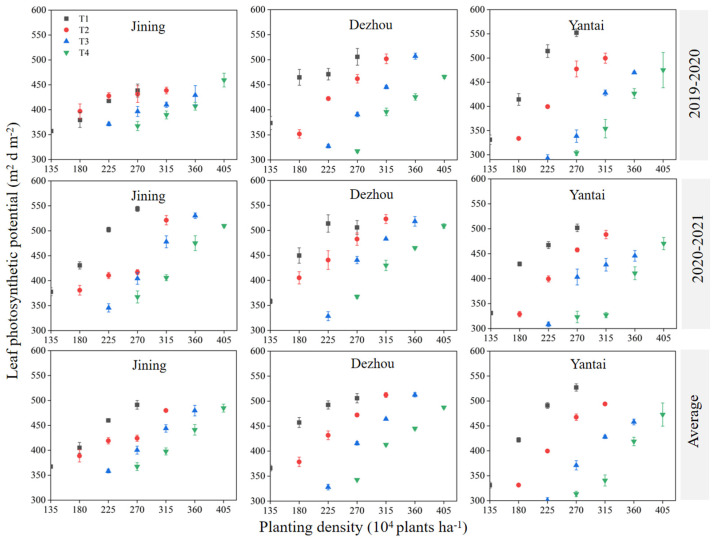
Effect of sowing time and density on leaf photosynthetic potential. Note: T1, T2, T3, and T4 indicate sowing on October 5, October 15, October 25, and November 5, respectively. Data are expressed as the mean of three replicates and vertical bars indicate standard deviation.

The trends in the LAD and MLAI over the two years were similar. Specifically, the LAD exhibited an overall decline as the sowing date was delayed at the same planting density, while it increased with higher planting density for the same sowing date (Figure 3). Overall, these results suggest that earlier sowing dates and higher planting densities are associated with larger average LAD values. On average, across the two years, the comparison of the maximum LAD for each sowing date revealed that the maximum LAD under T1 was 2.6%, 5.1%, and 5.5% higher than under T2, T3, and T4, respectively.

#### 3.1.3. Net Assimilation Rate

**Figure 4:**
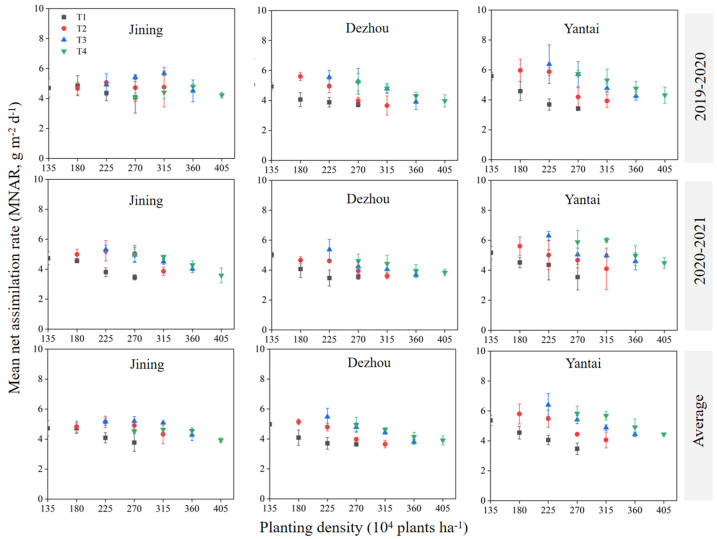
Effect of sowing time and density on mean net assimilation rate. Note: T1, T2, T3, and T4 indicate sowing on October 5, October 15, October 25, and November 5, respectively. Data are expressed as the mean of three replicates and vertical bars indicate standard deviation.

The trend in the MNAR exhibited an opposite pattern to that of the MLAI and LAD over the two years. The MNAR increased with later sowing dates at the same planting density, while it decreased with higher planting densities for the same sowing date (Figure 4). On average, the MNAR values across the experimental sites were as follows: Yantai (4.95 g m^−2^ d^−1^) > Jining (4.62 g m^−2^ d^−1^) > Dezhou (4.38 g m^−2^ d^−1^). Jining recorded the highest average MNAR over the two years on T3D4 (5.20 g m^−2^ d^−1^), while both Dezhou and Yantai achieved their highest averages on T3D3, with values of 5.48 g m^−2^ d^−1^ and 6.40 g m^−2^ d^−1^, respectively.

### 3.2. Biomass and Harvest Index

**Figure 5:**
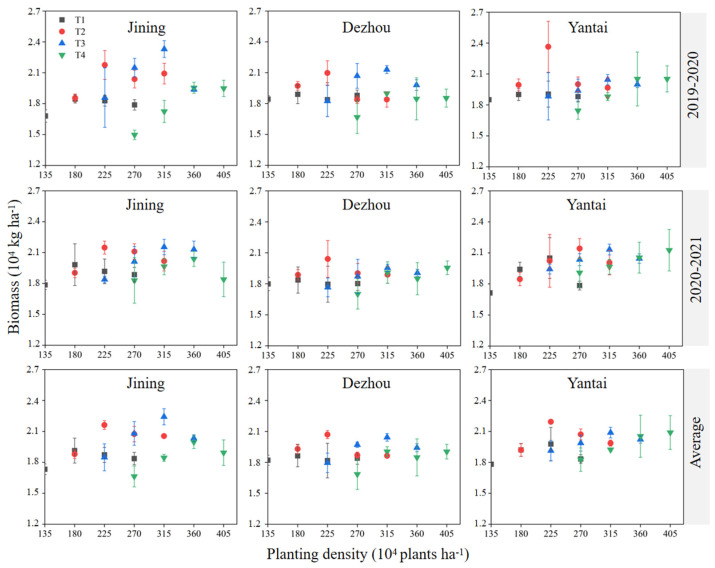
Effect of sowing time and density on biomass. Note: T1, T2, T3, and T4 indicate sowing on October 5, October 15, October 25, and November 5, respectively. Data are expressed as the mean of three replicates and vertical bars indicate standard deviation.

The biomass generally exhibited an initial increase followed by a decrease with the increase of planting density for the same sowing date. In contrast, the trend in biomass changes with sowing date was inconsistent at the same density (Figure 5). The maximum biomass was observed at a planting density of 225 × 10^4^ plants ha^−1^ on October 15 (T2D3) and 315 × 10^4^ plants ha^−1^ on October 25 (T3D5) in both the 2019–2020 and 2020–2021 periods.

**Figure 6:**
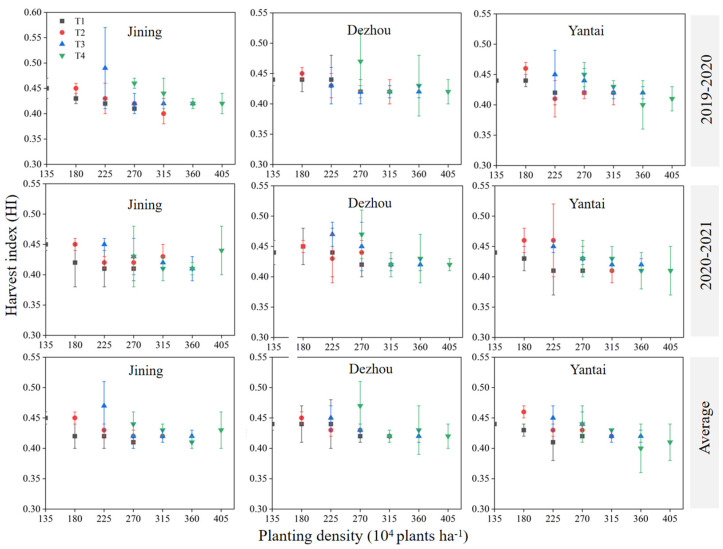
Effect of sowing time and density on harvest index (HI). Note: T1, T2, T3, and T4 indicate sowing on October 5, October 15, October 25, and November 5, respectively. Data are expressed as the mean of three replicates and vertical bars indicate standard deviation.

The harvest index (HI) typically decreased with increasing density under the same sowing date, although the trend in changes with sowing date varied at the same density (Figure 6). In two growing seasons, the maximum HI was achieved with planting 225 × 10^4^ plants ha^−1^ on October 25 (T3D3) in Jining, with planting 270 × 10^4^ plants ha^−1^ on Nov, 15 (T4D4) in Dezhou, and with planting 180 × 10^4^ plants ha^−1^ on October 15 (T2D2) in Yantai. No significant differences were found in the maximum HI values across the different sowing dates (*p* > 0.05).

### 3.3. Grain Yield and Composition Factors

#### 3.3.1. Grain Yield Components

**Figure 7:**
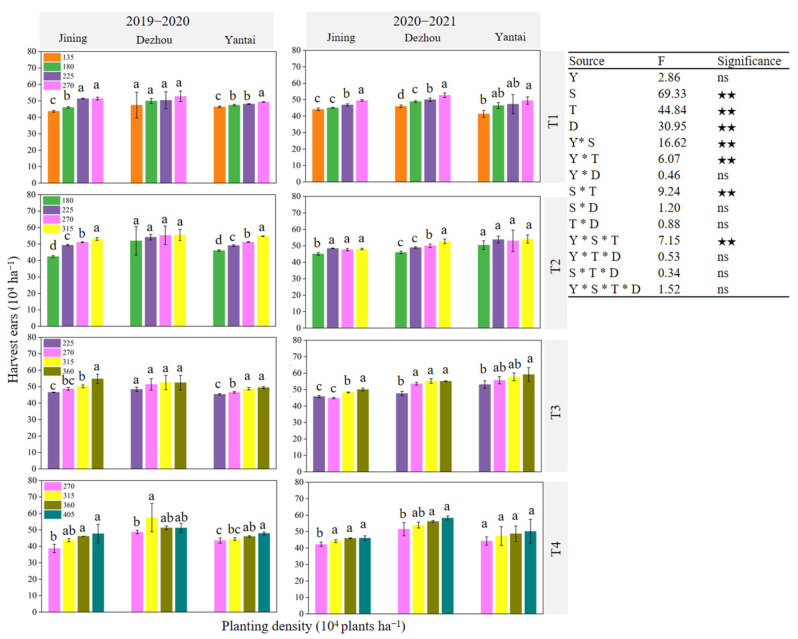
Effect of sowing time and density on number of harvested ears. Note: T1, T2, T3, and T4 indicate sowing on October 5, October 15, October 25, and November 5, respectively; Y: year; S: site; T: sowing date; D: density; ns: not significant; ★★: significant at *p* < 0.01. Data are expressed as the mean of three replicates and vertical bars indicate standard deviation. Different lowercase letters indicate significant differences among treatments at *p* < 0.05 using LSD method.

**Figure 8:**
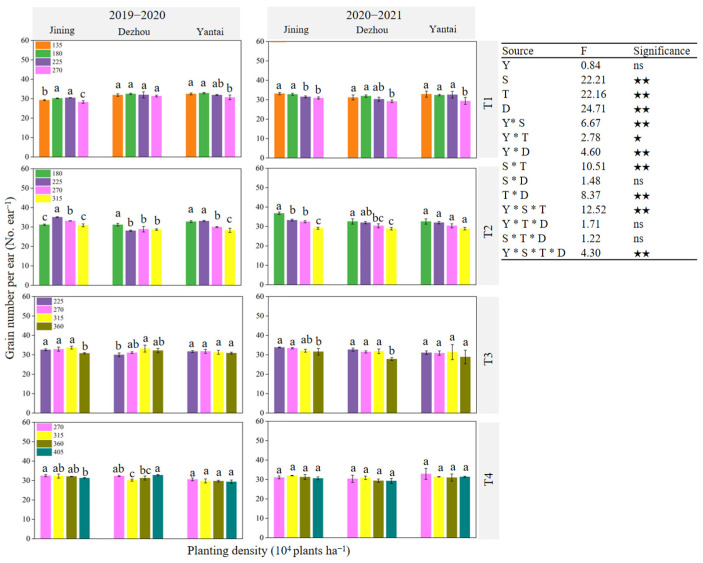
Effect of sowing time and density on grain number per ear. Note: T1, T2, T3, and T4 indicate sowing on October 5, October 15, October 25, and November 5, respectively; Y: year; S: site; T: sowing date; D: density; ns: not significant; ★: significant at *p* < 0.05; ★★: significant at *p* < 0.01. Data are expressed as the mean of three replicates and vertical bars indicate standard deviation. Different lowercase letters indicate significant differences among treatments at *p* < 0.05 using LSD method.

**Figure 9:**
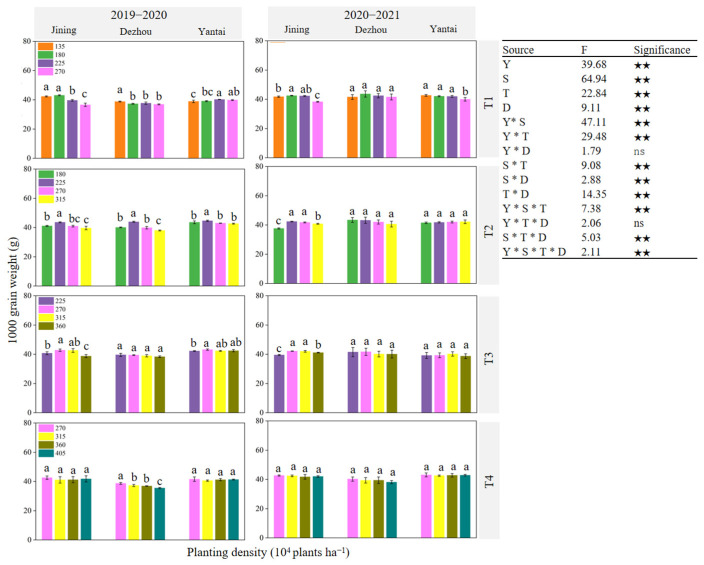
Effect of sowing time and density on 1000-grain weight. Note: T1, T2, T3, and T4 indicate sowing on October 5, October 15, October 25, and November 5, respectively; Y: year; S: site; T: sowing date; D: density; ns: not significant; ★★: significant at *p* < 0.01. Data are expressed as the mean of three replicates and vertical bars indicate standard deviation. Different lowercase letters indicate significant differences among treatments at *p* < 0.05 using LSD method.

Figure 7, Figure 8 and Figure 9 illustrate that the variation in the number of harvested ears was more pronounced with changes in the planting density than that of the number of grains per ear and grain weight. The parameters of the number of harvested ears, grains per ear, and thousand grain weight were significantly affected by the site, sowing date, and density (*p* < 0.01). Additionally, significant interactions were observed between the year and site, year and sowing date, site and sowing date, and year and site and sowing date, all of which influenced these parameters (*p* < 0.05). This indicated that the effect of the planting density on the number of harvested ears was greater than that on the number of grains per ear and grain weight. At each experimental site, the number of harvested ears generally increased under the same sowing date (Figure 7). The maximum average number of harvested ears over the two years was observed for T1 under D4, T2 under D5, T3 under D6, and T4 under D7. The trend in the grain number per ear varied across the experimental sites, sowing dates, and planting densities in the two-year experiment (Figure 8). For the thousand-grain weight, the highest values across the two years were achieved at D1 and D2 under T1, D3 under T2, D4 under T3, and D4 under T4 at the respective sites (Figure 9).

#### 3.3.2. Grain Yield

**Figure 10:**
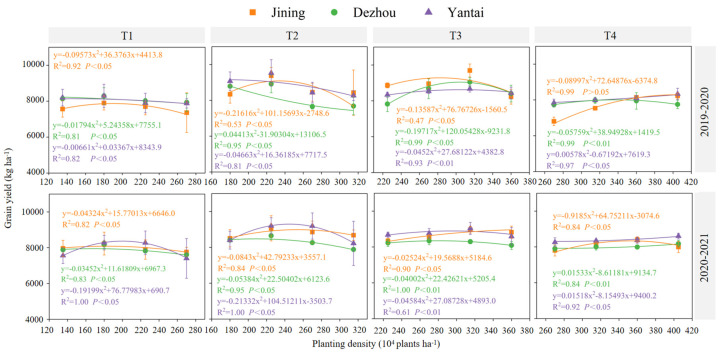
Effect of sowing time and density on yield. Note: T1, T2, T3, and T4 indicate sowing on October 5, October 15, October 25, and November 5, respectively. Data are expressed as the mean of three replicates and vertical bars indicate standard deviation.

The comparison between the years revealed that the yield in 2020–2021 was 2.3%, −0.6%, and 1.1% higher than that of 2019–2020 for Jining, Dezhou, and Yantai, respectively. On average over the two years, the yield in Yantai was 1.3% and 3.3% higher than in Jining and Dezhou (Figure 10). A binomial regression analysis was conducted on the grain yield at different planting densities under the same sowing date across the regions. The R^2^ and *p* values indicated a good fit, effectively representing the trend in grain yield variation with planting density (Figure 10). Overall, the grain yield of winter wheat showed a trend of initially increasing and then decreasing with a later sowing date or increasing density. As shown in Figure 10, D2 under T1, D3 under T2, D5 under T3, and D6 and D7 under T4 were most likely to achieve higher grain yields.

### 3.4. Quality Characteristics

#### 3.4.1. Quality

**Figure 11:**
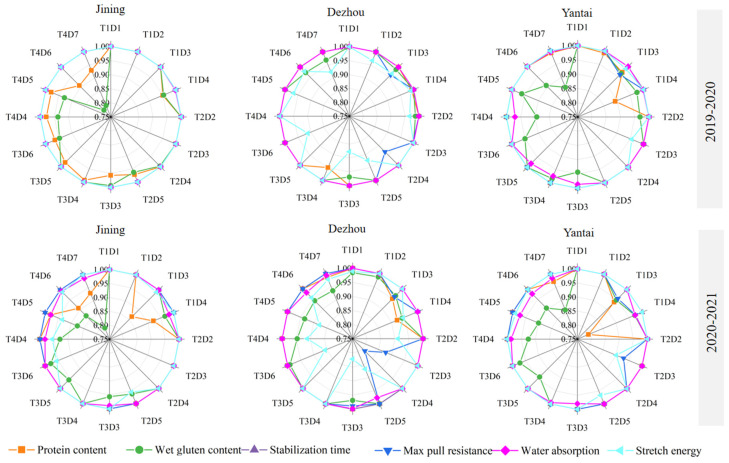
Effect of sowing time and density on quality parameters. Note: T1D1, T1D2, T1D3, T1D4, T2D2, T2D3, T2D4, T2D5, T3D3, T3D4, T3D5, T3D6, T4D4, T4D5, T4D6, and T4D7 refer to planting 135 × 10^4^ plants ha^−1^ on October 5, 180 × 10^4^ plants ha^−1^ on October 5, 225 × 10^4^ plants ha^−1^ on October 5, 270 × 10^4^ plants ha^−1^ on October 5, 180 × 10^4^ plants ha^−1^ on October 15, 225 × 10^4^ plants ha^−1^ on October 15, 270 × 10^4^ plants ha^−1^ on October 15, 315 × 10^4^ plants ha^−1^ on October 15, 225 × 10^4^ plants ha^−1^ on October 25, 270 × 10^4^ plants ha^−1^ on October 25, 315 × 10^4^ plants ha^−1^ on October 25 and 360 × 10^4^ plants ha^−1^ on October 25, 270 × 10^4^ plants ha^−1^ on Nov. 5, 315 × 10^4^ plants ha^−1^ on Nov. 5, 360 × 10^4^ plants ha^−1^ on Nov. 5, and 405 × 10^4^ plants ha^−1^ on Nov. 15.

The protein content compliance rate varied across the regions as follows: Dezhou (84.3%) > Yantai (78.1%) > Jining (53.1%) (Figure 11). The impact of the sowing date and density on wheat protein content was inconsistent across regions, and no consistent trends were observed across the different experimental years.

The difference in wet gluten content compliance rates between the regions was small, and the overall compliance rate was relatively low, ranging from 39% to 50%. Late sowing, regardless of the region or experimental year, did not promote an increase in the wet gluten content, and thus, it was not conducive to achieving the strong-gluten standard (Figure 11).

The compliance rates for water absorption across the regions were as follows: Dezhou (87.5%) > Jining (78.1%) > Yantai (59.3%). From 2019 to 2020, the water absorption rate in Jining met the national strong-gluten standard, except for the treatment with 270 × 10^4^ plants ha^−1^ on October 5 (T1D4). In Dezhou, all the treatments achieved the national strong-gluten standard. In both the 2019–2020 and 2020–2021 periods, all planting densities under T2 in Yantai achieved the strong-gluten standard for water absorption (Figure 11).

The dough rheological properties of Jimai 44 across the three regions indicated that it was generally easy to meet the strong-gluten standard. The quality ranking was follows: stable time (100%) > maximum tensile resistance (88.5%) > dough energy (67.7%) (Figure 11). In the two-year experiment in Jining, the stability time and maximum tensile resistance met the national strong-gluten standard. In Dezhou and Yantai, the maximum tensile resistance was higher with later sowing dates (T3, T4).

#### 3.4.2. Comprehensive Quality Compliance Rate

**Figure 12:**
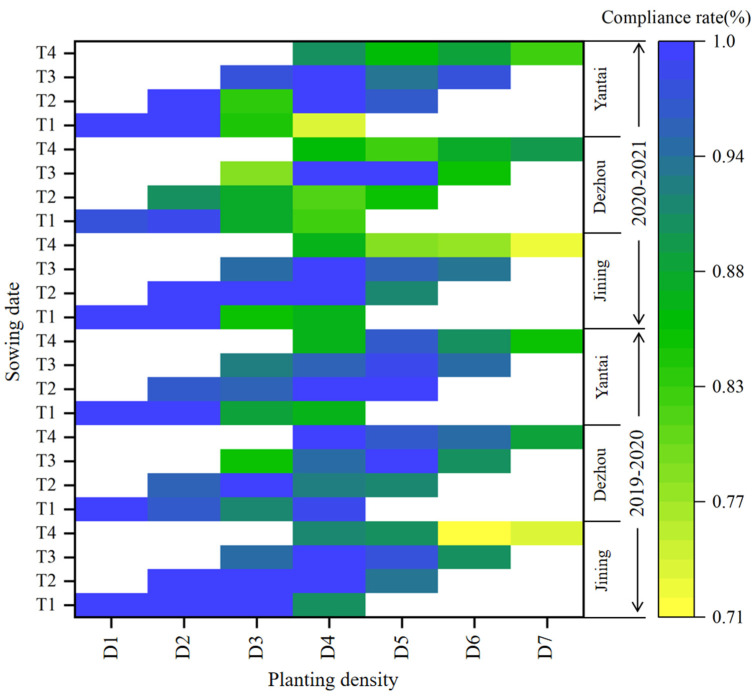
Analysis of sample quality compliance rate. Note: T1, T2, T3, and T4 indicate that the sowing date was October 5, October 15, October 25, and November 5, respectively; D1, D2, D3, D4, D5, D6, and D7 indicate that the planting density was 135, 180, 225, 270, 315, 360, 405 × 10^4^ plants ha^−1^, respectively.

The comprehensive quality compliance rate of a sample is the product of each standardized quality parameter. Figure 12 shows that the highest compliance rates were observed for D1 and D2 under T1; D2, D3, and D4 under T2; D4 and D5 under T3; and D4 under T4. The comprehensive quality compliance rate varied across the sowing dates in the following order: T2 > T3 > T1 > T4. The compliance rate under T4 was reduced by 7.6–9.0% compared to the other sowing dates. Among the different planting densities, the comprehensive quality compliance rate followed the order of D1 > D2 > D5 > D4 > D3 > D6 > D7.

### 3.5. Correlation Analysis

**Figure 13:**
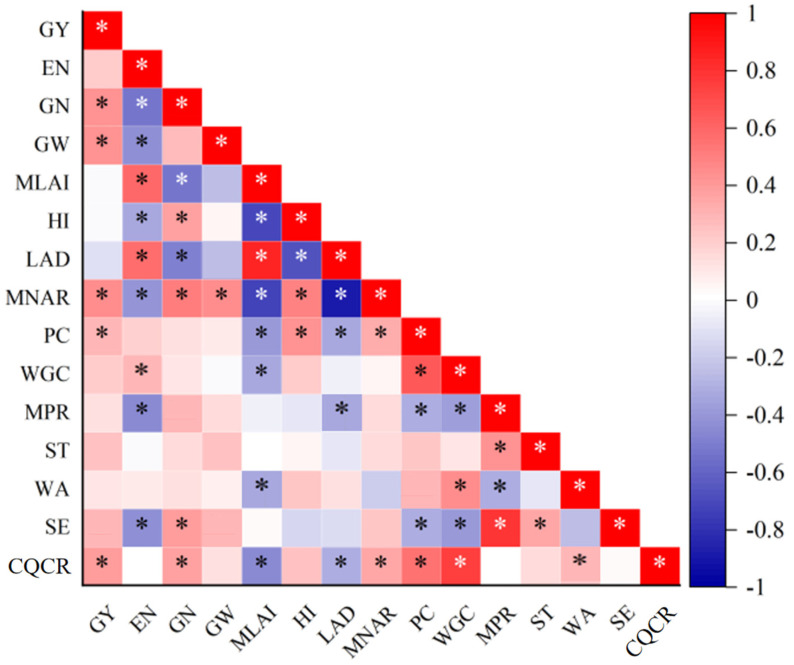
Relationship between yield and quality-related variables. Note: MLAI, mean leaf area index; MNAR, mean net assimilation rate; HI, harvest index; GY, yield; EN, number of ears; GN, number of grains per ear; GW, grain weight, PC, protein content; WGC, wet gluten content; ST, stabilization time; MPR, max pull resistance; WA, water absorption; SE, stretch energy; CQCR, comprehensive quality compliance rate. * denotes significance at the 0.05 probability level.

Figure 13 illustrates a significant positive correlation (*p* < 0.05) between the grain yield (GY) and several factors, including the number of grains per ear (GN), grain weight (GW), mean net assimilation rate (MNAR), protein content (PC), and comprehensive quality compliance rate (CQCR). Additionally, the GN and GW were significantly positively correlated with the MNAR (*p* < 0.05). The CQCR was positively correlated with yield traits (GY, number of harvested ears (EN), GN, and GW), the harvest index (HI), the MNAR, and quality traits, while exhibiting a significant negative correlation with the mean leaf area index (MLAI) and photosynthetic potential (LAD) (*p* < 0.05).

## 4. Discussion

### 4.1. The Influence of Sowing Date and Density on Leaf Assimilation Ability

The photosynthetically active radiation intercepted by the wheat canopy increases with the leaf area index (LAI) and photosynthetic potential (LAD), which are key indicators of wheat photosynthetic performance [22]. Both the LAI and LAD can be adjusted by modifying the sowing date and planting density [23]. In this study, under identical planting densities, the mean LAI and LAD decreased with delayed sowing (Figure 2 and Figure 3), a trend consistent with the findings of Chen et al. [22], who observed that the LAI in winter wheat decreased with delayed sowing dates. This reduction may be due to early sowing capturing more water and light, promoting better leaf growth and enhancing photosynthesis [24].

Conversely, under the same sowing date, both the LAI and LAD increased with an increase in planting density (Figure 2 and Figure 3). This suggests that a higher planting density intensifies the competition for resources (light, water, and nutrients), which may encourage plants to expand their leaf area to optimize light capture and their photosynthetic efficiency [23,25]. Therefore, in practical production, earlier sowing dates (T1 or T2) should be coupled with higher densities to maximize the MLAI and LAD. In contrast, when sowing is delayed (T4), higher densities (360–405 × 10^4^ plants ha^−1^) were necessary to maintain a higher MLAI and LAD. This study suggests that planting densities should not fall below D1, D2, D3, and D4, respectively, for each sowing date (T1–T4) to sustain the basic MLAI and LAD values.

Photosynthesis plays a critical role in crop yield formation, and enhancing photosynthetic efficiency is a key strategy for increasing yield [26]. The mean net assimilation rate (MNAR) is a key metric for evaluating photosynthetic capacity, and optimizing the MNAR can enhance the material production potential of crops [27]. Our study found that the maximum MNAR values under the different sowing dates followed a trend of initial increase and subsequent decrease with delayed sowing date (Figure 4), similar to the results of Shao et al. [28]. This pattern may be attributed to the negative effects of late sowing on assimilate transport and accumulation, particularly after flowering [28].

Furthermore, an increased planting density initially increased the MNAR but later led to a decline (Figure 4). Excessively late sowing and a high planting density were identified as major contributors to a reduced MNAR. In agricultural practice, late sowing should be avoided where possible to preserve the assimilation capacity, and high planting densities should be managed to prevent resource depletion and risks of lodging.

### 4.2. The Effect of Sowing Date and Density on Grain Yield and Related Indicators

Previous studies have demonstrated the significant impact of sowing date, planting density, and their interaction on wheat grain yield and its associated parameters [29]. This study corroborates these findings, with sowing date, planting density, and site all showing significant effects on yield components (*p* < 0.01). Additionally, significant interactions between experimental factors (year, site, and sowing date) were observed (*p* < 0.05), highlighting the complex relationship between environmental variables and wheat yield (Figure 13).

We observed that the grain yield of winter wheat increased with a higher planting density up to an optimal point before decreasing with further increases in density (Figure 10). This reduction in yield with early sowing may be attributed to excessive growth before overwintering, leading to higher nutrient consumption and susceptibility to freezing damage, which negatively affects the grain number and weight [30]. Delayed sowing, on the other hand, shortened the pre-winter growth period, resulting in weak seedlings, fewer tillers, and reduced cold tolerance, ultimately lowering the yield in the following season [31].

Consistent with the results of Zhang et al. [32], our results indicate that excessive planting density can reduce wheat yield by impairing flag leaf photosynthesis and aboveground biomass accumulation. Notably, the optimal planting densities (D2 under T1, D3 under T2, D5 under T3, and D6 under T4) led to the highest grain yields (Figure 10), while densities beyond these thresholds did not significantly improve the yields. Early sowing with lower densities can enhance physiological activity and prolong leaf lifespan, thus improving post-flowering grain filling and ultimately increasing yield [29].

### 4.3. The Effect of Sowing Date and Density on Wheat Grain Quality

Wheat grain quality is highly sensitive to cultivation practices and environmental conditions [26]. This study found that the variability in wheat quality was primarily driven by dough rheological properties (stability time, maximum tensile resistance, and dough energy), followed by the protein content and wet gluten content (Figure 11). As observed by Geleta et al. [33], low planting densities can enhance wheat quality traits, and this study found similar results, with the optimal protein and gluten contents achieved under early sowing at lower densities. This can be attributed to the relationship between the sowing date, density, and nutrient absorption, which influences photosynthetic performance and nutrient assimilation.

Furthermore, late sowing combined with a high density was detrimental to dough water absorption, in line with the findings from Chen et al. [34]. Our results showed a strong positive correlation between dough stability time, maximum tensile resistance, and dough energy [13]. Excessively early sowing and high-density planting, as well as excessively late sowing, were found to impair the comprehensive quality of wheat. Early sowing at lower densities (T2, 180–225 × 10^4^ plants ha^−1^) was found to improve the quality of strong-gluten wheat.

### 4.4. Limitations and Areas for Further Research

While this study provides valuable insights into the effects of sowing date and density on wheat yield and quality, several areas warrant further exploration. First, the physiological and ecological responses of different wheat varieties to varying sowing dates and densities should be studied to enhance the universality of these findings [35]. Second, long-term climate data, including historical and future projections, could provide a broader context for understanding the impacts of environmental variability on wheat performance [36].

Moreover, pest and disease management strategies were not included in this study, although they play a significant role in wheat yield and quality [37]. Integrating pest and disease control measures into future research could provide a more comprehensive understanding of wheat production systems. Additionally, interactions between the sowing date, density, and other agricultural management practices, such as fertilization and irrigation, need further investigation [38,39].

## 5. Conclusions

This study indicates that optimizing and coordinating the sowing time and planting density in various ecological regions can improve the assimilation ability of wheat leaves, promote biomass accumulation and its transport to grains, and ultimately synergistically improve grain yield and quality.

Based on our experimental results, we recommend the following planting strategies: (1) the optimal sowing time and density combination of D3 under T2 and D5 under T3 for Dezhou (northwest Shandong plain wheat region) and Jining (southwest Shandong plain and lake basin wheat region), and (2) D4 under T2 for Yantai (eastern and central Shandong mountainous and hilly wheat region) for a synergistic improvement in yield and quality of strong-gluten wheat. Our study underscores the importance of avoiding excessively early or late sowing times and high planting densities across the three major wheat-growing regions of Shandong Province. If late sowing becomes unavoidable due to environmental constraints, we recommend increasing the planting density to D5-D6 to maintain both the grain yield and quality, thereby mitigating agricultural production risks.

Furthermore, while this study emphasizes the synergistic effects of optimal sowing date and density combinations on winter wheat yield and quality, we acknowledge the necessity of integrating other cultivation practices such as fertilization, irrigation, and chemical control. Future research should focus on combining these factors to enhance wheat production and prevent the decline in yield and quality associated with late sowing or a high planting density. Continued investigation will be critical for optimizing cultivation management practices to improve wheat yield and quality, ultimately supporting sustainable wheat production.

## Figures and Tables

**Table 1:** Soil properties of 0–20 cm soil layer before the experiment at the experimental sites.

Site	Total Nitrogen	Available Nitrogen	Available Phosphorus	Available Potassium	Organic Matter
	(g·kg^−1^)	(mg·kg^−1^)	(mg·kg^−1^)	(mg·kg^−1^)	(g·kg^−1^)
Jining	1.46	89.1	54.2	117.8	11.1
Dezhou	1.38	81.2	50.1	127.4	10.6
Yantai	1.3	75.8	62.5	102.3	11.8

**Table 2:** Experimental treatments in 2019–2020 and 2020–2021.

Sowing Date	Density (D, 10^4^ plants ha^−1^)						
	135 (D1)	180 (D2)	225 (D3)	270 (D4)	315 (D5)	360 (D6)	405 (D7)
October 5 (T1)	T1D1	T1D2	T1D3	T1D4			
October 15 (T2)		T2D2	T2D3	T2D4	T2D5		
October 25 (T3)			T3D3	T3D4	T3D5	T3D6	
November 5 (T4)				T4D4	T4D5	T4D6	T4D7

## Data Availability

Data are contained within the article.
